# The association between renal hyperfiltration and mortality is not mediated by diabetes mellitus

**DOI:** 10.1007/s11255-023-03562-9

**Published:** 2023-03-23

**Authors:** Mounir Ould Setti, Ari Voutilainen, Leo Niskanen, Tomi-Pekka Tuomainen

**Affiliations:** 1 Institute of Public Health and Clinical Nutrition, University of Eastern Finland, Yliopistonranta 1., 70210 Kuopio, Finland; 2 Global Database Studies, IQVIA, Espoo, Finland; 3 Department of Internal Medicine, Päijät-Häme Central Hospital, Lahti, Finland

**Keywords:** Mortality, Diabetes, Glomerular filtration rate, Renal hyperfiltration, Heart disease risk factors

## Abstract

**Background:**

Renal hyperfiltration (RHF), recently established as a risk factor for mortality, is linked to current and subsequent diabetes mellitus (DM). DM could be seen as a mediator in the pathway between RHF and mortality. However, the mediating role of DM in the relationship between RHF and mortality is unclear.

**Methods and results:**

Based on a cohort of 2682 Finnish men from the Kuopio Ischaemic Heart Disease Risk Factor Study (KIHD) followed-up for 35 years, we evaluated the association between RHF and mortality, with DM as a mediator, following two methods: a classic mediation analysis approach, using Cox regression, and a counterfactual framework for mediation analysis, using g-computation, Cox regression, and logistic regression. RHF is associated with an increased risk of mortality. This association was not mediated by DM. Under a counterfactual framework and on a hazard ratio scale, RHF association with mortality had a total effect of 1.54 (95% confidence interval, 1.26–1.98) and a controlled direct effect of 1.66 (1.34–2.16).

**Conclusion:**

An association between RHF and mortality risk, independent of DM, was established. RHF should be considered, managed, and followed-up as a mortality-associated condition, regardless of the status of DM. We suggest clinicians to consider including RHF screening in routine clinical care, especially diabetic care.

**Supplementary Information:**

The online version contains supplementary material available at 10.1007/s11255-023-03562-9.

## Introduction

Abnormal increase in glomerular filtration rate (GFR), termed renal hyperfiltration (RHF), was mainly regarded as an early sign of kidney damage in diabetes mellitus (DM), especially type 1 [1–3]. While RHF is prevalent in diabetes mellitus (DM), with figures as high as 75% in type 1 DM [4] and 40% in type 2 DM [4–6], RHF was recently established as a risk factor for mortality [7], both cardiovascular [8] and non-cardiovascular [9, 10], in an apparently healthy population, unconstrained by DM [11–13]. RHF is linked to subsequent diabetes mellitus (DM). For instance, RHF could be a precursor to DM, manifesting in prediabetes [14]. In addition, RHF is linked to chronic kidney disease (CKD) [15]. Among nearly 17,000 individuals from a cohort study [16], those with RHF had 8.7 times (95% CI: 4.2–18.1) higher risk for a rapid decline of estimated GFR (eGFR), impending an increased risk of CKD. Whilst DM is the most common cause of CKD [17], RHF was particularly associated with diabetic kidney disease, in a meta-analysis by Magee et al. [18]. Further, among patients with DM, those with RHF were at a higher risk of mortality [13]. Concurrently, DM is well established as a risk factor for mortality [19].

DM could be seen as a mediator in the pathway between RHF and mortality. While the link between RHF and mortality was assessed in both diabetic [13] and nondiabetic populations [11, 20], the direct effect of RHF on mortality, independently of future DM, is unclear. Using mediation analysis [21], we aimed through this study on evaluating the mediating role of DM in the relationship between RHF and mortality.

## Methods

### Data source and study population

The study is based on the Kuopio Ischaemic Heart Disease Risk Factor Study (KIHD), a cohort of 2682 men randomly sampled from the population of the region of Kuopio, Finland, between March 1984 and 1989 [22]. The cohort is linked to the Finnish Care Register for Healthcare (HILMO) (License THL/93/5.05.00/2013) and to the cause-of-death registry of Finland (License TK-53-1770-16), among other registries.

After excluding 50 patients with baseline CKD (eGFR ≤ 60 ml/min/1.73 m^2^) and 258 men with missing values, we excluded 321 men who were abstinent from drinking alcohol at baseline, since they represented a special case differing from the study population in terms of education and socioeconomic status, health behaviors, and overall health [23]. Finally, we excluded 9 men with outlying values of body mass index (BMI), and weekly alcohol consumption, settling for a final study population of 2044 men, followed for a maximum of 35 years (median, 28 years). There was no loss to follow-up in our study.

### Variable measurement

Study participants were examined by a physician and a nurse who measured the men’s height, weight, and blood pressure, interviewed them and collected blood samples from them [19]. The men’s medical history, medications, and health behaviors were assessed through interviews and detailed structured questionnaires. Dietary intake was assessed through instructed 4-day food recording.

The study exposure of interest, RHF, was defined as eGFR values above the 95^th^ age-adjusted percentile within the study population. We computed eGFR using the Chronic Kidney Disease Epidemiology Collaboration (CKD-Epi) equation [24], based on Jaffe-corrected [25] serum creatinine values. Follow-up DM (International Statistical Classification of Diseases and Related Health Problems 10^th^ Revision [ICD-10] codes E10-E14) diagnosis was obtained through linkage to HILMO, serving as the study’s mediator of interest. For sensitivity analysis, a 20-year examination also assessed the survivors’ status of DM through interviews on medical and medication history and blood sample analysis for a serum glucose level.

We considered the following variables measured at baseline as covariates in our analyses: age, BMI [26] (as a categorical variable), hypertension status [27] (medication or medical history of hypertension or an elevated mean systolic (≥ 140 mm Hg) or diastolic (≥ 90 mm Hg) blood pressure), smoking status [28, 29] (current-, previous-, or never smoker), alcohol consumption [30] (grams per week), vitamin D level [31] (indicated by serum 25(OH)D_3_ [32], 25th percentile within the study population [29.02 ng/mL] as the cutoff between low and normal vitamin D levels), and the healthy Nordic diet (HND) score [33] (based on the Baltic Sea Diet Score, a validated indicator of diet quality in the Nordic countries [34]).

The outcome of our study, mortality due to any cause, was sourced through the Finnish cause-of-death registry and ascertained using the Finnish personal identification code.

### Data analysis

**Fig. 1:**
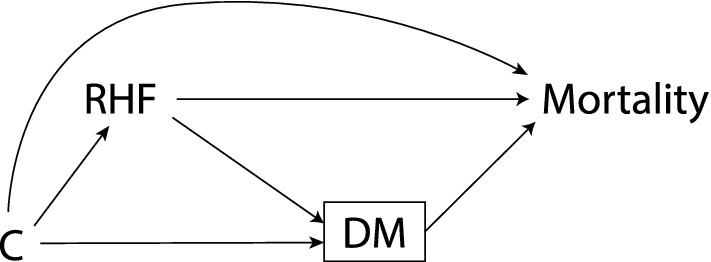
Directed acyclic graph illustrating the relation between the exposure of interest, renal hyperfiltration (RHF), the mediator, diabetes mellitus (DM), the outcome of interest, mortality, and potential confounders (C). The box around DM suggests that conditioning was done on this variable (through restriction)

First, we described the study population in terms of baseline characteristics and occurrence of DM during follow-up, with comparisons between survivors and non-survivors, using Chi-square, Kruskal–Wallis, and Mann-Whitey *U* tests. Then, in a classic mediation analysis approach [35], we examined the hazard ratio (HR) of the association between RHF and mortality, with DM as the mediator (Fig. 1), in a. all the study population (no adjustment for baseline or follow-up DM) and b. excluding those who had a DM diagnosis at baseline or during follow-up. These HRs were estimated using Cox proportional hazard models, with a period at risk from baseline until the occurrence of the outcome or the last day of follow-up on 31 December 2018, crude (age-adjusted only) and adjusted for the following baseline covariates: age, BMI, hypertension, smoking status, alcohol consumption, vitamin D level, and HND score. This approach assumes that there is no confounding between the RHF and DM, RHF and mortality, and DM and mortality, and that there is no interaction between RHF and DM, and that no variable confounding the relation between DM and mortality is affected by RHF. A relation of mediation would be suggested if the effect of RHF on mortality would disappear after adjustment for the mediator.

**Fig. 2:**
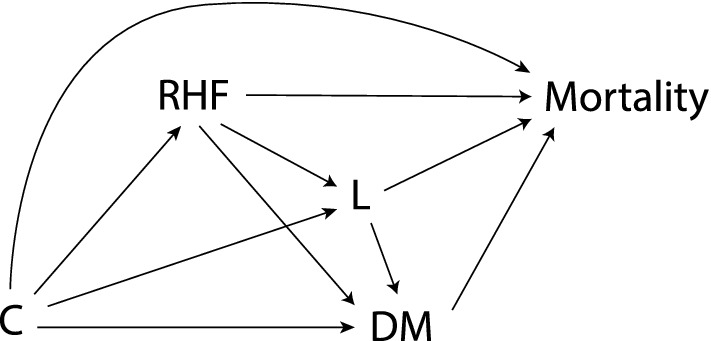
Directed acyclic graph illustrating the relation between the exposure of interest, renal hyperfiltration (RHF), the mediator, diabetes mellitus (DM), the outcome of interest, mortality, and potential confounders (C). L represents an unmeasured exposure-induced mediator-outcome confounder

Third, to better examine the mediating role of DM in the RHF-mortality relationship, we performed a mediation analysis, in a counterfactual framework [36, 37], using non-parametric g-computation [38, 39], with RHF as the exposure, DM as the mediator, and mortality as the outcome (Fig. 2). This approach is justified considering that conditioning on the mediator, as per the classic approach, may create a situation of collider bias (Fig. 2), confounding the relation between exposure and outcome [40, 41]. Additionally, considering that DM is associated with mortality through multiple pathways and that the long-term consequences of RHF are not well explored, unmeasured variables (L, Fig. 2) could be a source of exposure-induced confounding between the mediator and the outcome. In addition, our approach permits consideration for potential RHF-DM interactions [37].

We regressed the outcome variable using a Cox proportional hazard model and the mediator using logistic regression. We adjusted our models for age, DM, BMI, smoking, hypertension, alcohol consumption, vitamin D level, and HND score, all measured at baseline. Applying the g-formula [38], we used direct counterfactual imputation as the estimation method of the causal effects, expressed in HRs, and bootstrapping to obtain their 95% confidence intervals [42]. We decomposed the total RHF effect on mortality considering direct, indirect, and pure natural effects [43, 44].

Finally, as a sensitivity analysis (SA) of the classic mediation analysis, SA1. We included in the fully adjusted Cox regression model examining the association between RHF and mortality in all study population an interaction term between RHF and baseline DM. As SAs of the mediation analysis performed according to the counterfactual framework, SA2. We used KIHD diagnoses of DM at 20 years of follow-up as the mediator instead of the diagnosis collected via linkage with HILMO throughout the follow-up, and SA3. We used baseline DM instead of follow-up DM as a mediator.

All computations were performed by means of R version 4.2.2 (https://www.R-project.org).

## Results

At the end of follow-up, the study outcome, mortality, occurred in 1289 men out of 2044 (63.06%). In comparison to the survivors, non-survivors had a higher proportion of RHF (7.21 versus [vs.] 2.65%, *p* < 0.001), a higher proportion of baseline DM (8.15 vs. 2.38%, *p* < 0.001), and a higher proportion of follow-up DM (15.59 vs. 11.52%; *p* < 0.001). Descriptive statistics of the study population’s baseline and follow-up characteristics by mortality outcome are described in Table 1.

**Table 1:** Baseline characteristics and follow-up differences by mortality outcome

	Overall	Survivors	Non-survivors	*P* value*
*N* (%)	2044 (100.00)	755 (36.94)	1289 (63.06)	
Age in years	54.33 [48.92, 54.50]	54.17 [48.08, 54.42]	54.42 [54.25, 54.75]	< 0.001
BMI (column %)				< 0.001
≤ 25	647 (31.65)	293 (38.81)	354 (27.46)	
(25, 27.5]	634 (31.02)	241 (31.92)	393 (30.49)	
(27.5, 30]	414 (20.25)	128 (16.95)	286 (22.19)	
(30, 32.5]	218 (10.67)	64 (8.48)	154 (11.95)	
> 32.5	131 (6.41)	29 (3.84)	102 (7.91)	
Smoking status (column %)				< 0.001
Never smoker	587 (28.72)	312 (41.32)	275 (21.33)	
Previous smoker	748 (36.59)	272 (36.03)	476 (36.93)	
Current smoker	709 (34.69)	171 (22.65)	538 (41.74)	
Hypertension (%)	1216 (59.49)	380 (50.33)	836 (64.86)	< 0.001
Vitamin D deficiency** (%)	488 (23.87)	151 (20.00)	337 (26.14)	0.002
DM (column %)	123 (6.02)	18 (2.38)	105 (8.15)	< 0.001
eGFR in ml/min/1.73 m^2^	85.99 [77.25, 96.77]	84.86 [76.93, 95.36]	86.34 [77.50, 96.89]	0.072
RHF (column %)	113 (5.53)	20 (2.65)	93 (7.21)	< 0.001
Follow-up DM (column %)	288 (14.09)	87 (11.52)	201 (15.59)	0.013
Follow-up in years	27.84 [18.61, 31.24]	31.69 [30.24, 32.89]	21.62 [13.17, 26.93]	< 0.001
Age of death in years	78.79 [72.12, 85.05]	84.33 [78.48, 87.74]	75.33 [67.52, 82.21]	< 0.001

Characteristics concern variables measured at baseline, unless otherwise indicated. Numbers indicate median [interquartile range], unless otherwise indicated

*BMI* body mass index in kg/m^2^, *DM* diabetes mellitus, *eGFR* estimated glomerular filtration rate, *RHF* renal hyperfiltration

*Kruskal–Wallis’ rank-sum test and the Chi-square test were used for across-groups comparisons

**Serum 25(OH)D3 level lower or equal to the population’s 10th percentile, corresponding to values ≤ 22.1 ng/mL

In the classic mediation analysis, the HR of the total effect of RHF on mortality, with no adjustment for DM, was 1.83 (95% CI 1.48–2.26) in the crude Cox regression model and 1.56 (1.26–1.94) in the adjusted model, when compared to normal eGFR. When restricting the Cox regression analysis to those who did not have DM at baseline or during follow-up, RHF association with mortality presented with an HR of 1.88 (1.50–2.37) in the crude model and 1.57 (1.24–1.99) in the adjusted model.

**Fig. 3:**
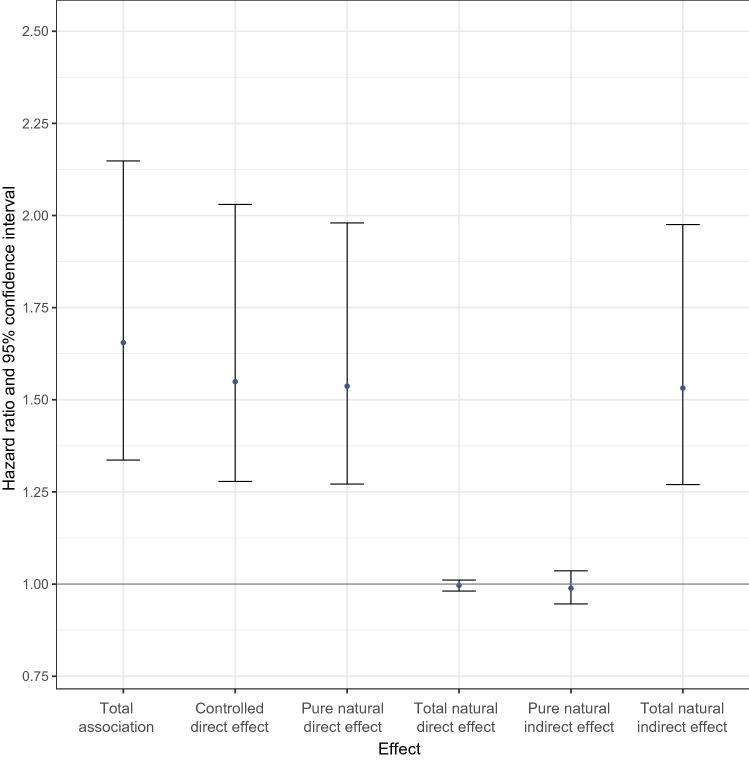
Effect decomposition of the mediation analysis using a counterfactual framework. Total Association: refers to the total effect of renal hyperfiltration on mortality, including both direct and indirect pathways. Controlled Direct Effect: refers to the effect of renal hyperfiltration on mortality that remains after controlling for the effect of the mediator. Pure Natural Direct Effect: refers to the effect of renal hyperfiltration on mortality when the mediator is set at the level that would naturally be observed in the absence of renal hyperfiltration. Total Natural Direct Effect: refers to the effect of renal hyperfiltration on mortality when the mediator is set at the level that would naturally be observed in the presence of renal hyperfiltration. Pure Natural Indirect Effect: captures the effect of the mediator on mortality in the absence of renal hyperfiltration. Total Natural Indirect Effect: captures the effect of the mediator on mortality in the presence of renal hyperfiltration

In the mediation analysis under a counterfactual framework, the effect decomposition of RHF on mortality showed, on an HR scale, a total effect of 1.54 (95% CI 1.26–1.98), a controlled direct effect of 1.66 (1.34–2.16), a total natural direct effect of 1.54 (1.27–1.98) and a total natural indirect effect of 0.98 (0.95—1.04). The overall proportion of the effect of RHF on mortality that was mediated by DM was minimal (− 5%, *p*-value=0.682) (Table 2 and Fig. 3, full details in Supplementary material).

**Table 2:** Effect decomposition of the relation between renal hyperfiltration and mortality, considering follow-up diabetes mellitus as the mediator, on the hazard ratio scale, using a counterfactual framework for mediation analysis

	Effect estimate*	95% CI lower limit	95% CI upper limit	*P*-value
Total association	1.54	1.26	1.98	< 0.001
Controlled direct effect	1.66	1.34	2.16	< 0.001
Pure natural direct effect	1.57	1.26	2.00	< 0.001
Total natural direct effect	1.54	1.27	1.98	< 0.001
Pure natural indirect effect	0.99	0.98	1.01	0.668
Total natural indirect effect	0.98	0.95	1.04	0.682
Overall proportion mediated, %	− 5	− 19	10	0.682
Overall proportion attributable to interaction, %	− 25	− 60	8	0.152

Total Association: refers to the total effect of renal hyperfiltration on mortality, including both direct and indirect pathways. Controlled Direct Effect: refers to the effect of renal hyperfiltration on mortality that remains after controlling for the effect of the mediator. Pure Natural Direct Effect: refers to the effect of renal hyperfiltration on mortality when the mediator is set at the level that would naturally be observed in the absence of renal hyperfiltration. Total Natural Direct Effect: refers to the effect of renal hyperfiltration on mortality when the mediator is set at the level that would naturally be observed in the presence of renal hyperfiltration. Pure Natural Indirect Effect: captures the effect of the mediator on mortality in the absence of renal hyperfiltration. Total Natural Indirect Effect: captures the effect of the mediator on mortality in the presence of renal hyperfiltration

*CI* confidence interval

*Data are presented as hazard ratios, unless otherwise indicated

In the SA of the classic mediation analysis (SA1), the HR of the association between RHF and mortality remained relatively consistent on the inclusion of an interaction term between RHF and baseline DM, which the HR did not show an association with mortality. The SA results related to the mediation analysis in a counterfactual framework also remained relatively consistent with the main analysis (Supplementary material).

## Discussion

In a population-based cohort of middle-aged Finnish men with a 35-year follow-up, RHF associated with an increased risk of mortality. This association was not mediated by DM. These results were consistent using a classic mediation analysis approach, in both crude and adjusted models, and using a counterfactual framework, applying the g-formula. In addition, no interaction between RHF and DM regarding mortality was found.

In accordance with previous studies [7, 12], our results found an association between RHF and mortality risk independently of DM. However, to the best of our knowledge, our study is the first to consider the mediating role of follow-up DM in the relation between RHF and mortality. While the mechanism by which RHF associates with death is still unclear, especially that both cardiovascular and non-cardiovascular mortality are concerned, RHF should be considered, managed, and followed-up as a mortality-associated condition, regardless of the status of DM. Particularly in DM, RHF should benefit from special care to limit its extent and consequences. As Penno et al. [13] noted, DM patients with RHF are at a higher mortality risk than DM patients without RHF. In DM, an increase in GFR follows the increase in kidney size and tubular growth secondary to DM-associated hyperglycemia and obesity. In addition, hyperglycemia-induced upregulation of sodium-glucose cotransporters and proximal tubular sodium and glucose reabsorption increases single-nephron GFR by reducing afferent arteriolar resistance [45]. Consequently, glycemic control and weight loss could be efficient measures to reduce the harms of both DM and RHF. Nevertheless, the role of other measures, such as sodium-glucose cotransporter inhibitors [46] and dietary protein intake [47], in controlling RHF, also remains to be assessed. Finally, we suggest clinicians to consider including RHF screening in routine clinical care, especially diabetic care, and the research community to further profile RHF, despite its transient nature, as a disease, and to evaluate its public health burden.

Our study is strong by its long follow-up time, reliable exposure, mediators, covariates, and outcome assessment, and by its advanced and comprehensive methodology, including a number of sensitivity analyses, to which our findings remained consistent. However, multiple limitations could be counted.

First, the generalizability of our results is limited to middle-aged Finnish men. RHF might have a distinct pathogenesis and mortality profile in women [48]. Second, the ephemeral nature and the inter-day and intraday variations of GFR suggest that a single measure at baseline might not be sufficient to define RHF. Though additional measures at baseline could help ascertain exposure, the study design could benefit from consideration for time-varying exposure, throughout the follow-up.

IN addition, eGFR is not a perfect measure of GFR. While we corrected our serum creatinine measurement for the Jaffe assay [25], it is possible that this last might contribute to the overestimation of RHF in patients with increased serum glucose [49]. Serum cystatin C could be a better alternative to estimate GFR in patients with DM, when assessing RHF. Finally, further adjustment for grip strength [50] and central obesity [51] could improve our estimates, but it is unlikely to impact our findings which were consistent across crude and adjusted analyses.

## Clinical significance

Renal hyperfiltration has mostly been regarded under the lens of diabetes mellitus, although it is getting established as an independent risk factor for mortality. Often associated with prediabetes and presenting as a precursor of diabetes mellitus, renal hyperfiltration is linked to subsequent diabetes mellitus. Diabetes mellitus could, thus, be seen as a mediator in the pathway between renal hyperfiltration and mortality. However, the mediating role of diabetes mellitus in the association between renal hyperfiltration and mortality has not been studied. We found an association between renal hyperfiltration and mortality risk. The association was not mediated by diabetes mellitus. Renal hyperfiltration should be assessed and managed as an independent condition, regardless of the status of diabetes mellitus.

## Supplementary Information

Below is the link to the electronic supplementary material.

Supplementary file1 (PDF 80 KB)

## Data Availability

The University of Eastern Finland can be approached for requests of access to the KIHD dataset.
