# Supplementary material for: The association between renal hyperfiltration and mortality is not mediated by diabetes mellitus
Source: Int Urol Nephrol. 2023 Mar 23;55(10):2639–45. doi: 10.1007/s11255-023-03562-9 (PMC10499930; doi:10.1007/s11255-023-03562-9)
Supplement: Supplementary file 1 — Supplementary file1 (PDF 80 KB) [file 11255_2023_3562_MOESM1_ESM.pdf]

Main analysis Effect decomposition of the relation between renal hyperfiltration and mortality, considering follow-up diabetes mellitus as the mediator, on the hazard ratio scale, using a counterfactual framework for mediation analysis

	Effect estimate	Standard error	95% CI lower limit	95% CI upper limit	P-value
**Rcde**	1.66	0.20	1.34	2.16	<2e-16
**Rpnde**	1.56	0.18	1.27	2.01	<2e-16
**Rtnde**	1.54	0.18	1.27	1.98	<2e-16
**Rpnie**	0.99	0.01	0.98	1.01	0.668
**Rtnie**	0.98	0.02	0.95	1.04	0.682
**Rte**	1.54	0.18	1.26	1.98	<2e-16
**ERcde**	0.67	0.21	0.35	1.19	<2e-16
**ERintref**	-0.11	0.08	-0.26	0.05	0.152
**ERintmed**	-0.02	0.03	-0.07	0.05	0.716
**ERpnie**	-0.01	0.01	-0.02	0.01	0.668
**ERcde(prop)**	1.26	0.18	0.92	1.63	<2e-16
**ERintref(prop)**	-0.20	0.15	-0.53	0.08	0.152
**ERintmed(prop)**	-0.04	0.06	-0.15	0.09	0.716
**ERpnie(prop)**	-0.01	0.03	-0.04	0.02	0.668
**pm**	-0.05	0.07	-0.19	0.10	0.682
**int**	-0.25	0.17	-0.60	0.08	0.152
**pe**	-0.26	0.18	-0.62	0.08	0.152

CI, confidence interval; Rcde, controlled direct effect hazard ratio; Rpnde, pure natural direct effect hazard ratio; Rtnde, total natural direct effect hazard ratio; Rpnie, pure natural indirect effect hazard ratio; Rtnie, total natural indirect effect hazard ratio; Rte, total effect hazard ratio; ERcde, excess relative hazard due to controlled direct effect; ERintref, excess relative hazard due to reference interaction; ERintmed, excess relative hazard due to mediated interaction; ERpnie, excess relative hazard due to pure natural indirect effect; ERcde(prop), proportion ERcde; ERintref(prop), proportion ERintref; ERintmed(prop), proportion ERintmed; ERpnie(prop), proportion ERpnie; pm, overall proportion mediated; int, overall proportion attributable to interaction; pe, overall proportion eliminated

Sensitivity analysis 1 Hazard ratios of the association between renal hyperfiltration (RHF) and mortality after inclusion of an interaction term between RHF and baseline diabetes mellitus (bDM) in the fully adjusted Cox regression model assessing all study population (classic mediation analysis)

	Hazard Ratio	95% CI lower limit	95% CI upper limit
**RHF**	1.60	1.28	2.01
**bDM**	1.86	1.50	2.30
**RHF:bDM (Interaction)**	0.63	0.30	1.32

Sensitivity analysis 2 Effect decomposition of the relation between renal hyperfiltration and mortality, considering 20-year examination diabetes mellitus disease as the mediator, on the hazard ratio scale, using a counterfactual framework for mediation analysis

	Effect estimate	Standard error	95% CI lower limit	95% CI upper limit	P-value
**Rcde**	1.60	0.19	1.28	2.03	<2e-16
**Rpnde**	1.59	0.19	1.27	2.01	<2e-16
**Rtnde**	1.58	0.19	1.26	2.00	<2e-16
**Rpnie**	0.99	0.01	0.97	1.02	0.636
**Rtnie**	0.98	0.02	0.95	1.02	0.638
**Rte**	1.56	0.19	1.26	2.00	<2e-16
**ERcde**	0.61	0.20	0.29	1.06	<2e-16
**ERintref**	-0.03	0.02	-0.07	0.02	0.308
**ERintmed**	-0.02	0.02	-0.05	0.02	0.668
**ERpnie**	-0.01	0.01	-0.03	0.02	0.636
**ERcde(prop)**	1.10	0.10	0.95	1.25	<2e-16
**ERintref(prop)**	-0.05	0.05	-0.12	0.05	0.308
**ERintmed(prop)**	-0.03	0.04	-0.10	0.04	0.668
**ERpnie(prop)**	-0.02	0.03	-0.07	0.03	0.636
**pm**	-0.05	0.06	-0.17	0.06	0.638
**int**	-0.08	0.08	-0.20	0.05	0.308
**pe**	-0.10	0.10	-0.25	0.05	0.326

CI, confidence interval; Rcde, controlled direct effect hazard ratio; Rpnde, pure natural direct effect hazard ratio; Rtnde, total natural direct effect hazard ratio; Rpnie, pure natural indirect effect hazard ratio; Rtnie, total natural indirect effect hazard ratio; Rte, total effect hazard ratio; ERcde, excess relative hazard due to controlled direct effect; ERintref, excess relative hazard due to reference interaction; ERintmed, excess relative hazard due to mediated interaction; ERpnie, excess relative hazard due to pure natural indirect effect; ERcde(prop), proportion ERcde; ERintref(prop), proportion ERintref; ERintmed(prop), proportion ERintmed; ERpnie(prop), proportion ERpnie; pm, overall proportion mediated; int, overall proportion attributable to interaction; pe, overall proportion eliminated

Sensitivity analysis 3 Effect decomposition of the relation between renal hyperfiltration and mortality, considering baseline diabetes mellitus as the mediator, on the hazard ratio scale, using a counterfactual framework for mediation analysis

	Effect estimate	Standard error	95% CI lower limit	95% CI upper limit	P-value
**Rcde**	1.62	0.19	1.30	2.05	<2e-16
**Rpnde**	1.53	0.19	1.24	1.98	<2e-16
**Rtnde**	1.48	0.20	1.18	1.94	<2e-16
**Rpnie**	1.04	0.03	0.98	1.10	0.33
**Rtnie**	1.00	0.03	0.97	1.07	0.924
**Rte**	1.53	0.19	1.24	1.98	<2e-16
**ERcde**	0.59	0.18	0.29	0.98	<2e-16
**ERintref**	-0.05	0.08	-0.14	0.17	0.53
**ERintmed**	-0.04	0.05	-0.12	0.07	0.58
**ERpnie**	0.04	0.03	-0.02	0.10	0.33

**ERcde(prop)**	1.10	0.20	0.69	1.46	<2e-16
**ERintref(prop)**	-0.10	0.17	-0.41	0.23	0.53
**ERintmed(prop)**	-0.07	0.10	-0.29	0.10	0.58
**ERpnie(prop)**	0.07	0.08	-0.05	0.25	0.33
**pm**	0.00	0.06	-0.11	0.17	0.924
**int**	-0.17	0.23	-0.64	0.28	0.53
**pe**	-0.10	0.20	-0.46	0.31	0.664

CI, confidence interval; Rcde, controlled direct effect hazard ratio; Rpnde, pure natural direct effect hazard ratio; Rtnde, total natural direct effect hazard ratio; Rpnie, pure natural indirect effect hazard ratio; Rtnie, total natural indirect effect hazard ratio; Rte, total effect hazard ratio; ERcde, excess relative hazard due to controlled direct effect; ERintref, excess relative hazard due to reference interaction; ERintmed, excess relative hazard due to mediated interaction; ERpnie, excess relative hazard due to pure natural indirect effect; ERcde(prop), proportion ERcde; ERintref(prop), proportion ERintref; ERintmed(prop), proportion ERintmed; ERpnie(prop), proportion ERpnie; pm, overall proportion mediated; int, overall proportion attributable to interaction; pe, overall proportion eliminated
